# Antibiotic resistance in the most unlikeliest of places

**DOI:** 10.1111/1751-7915.12868

**Published:** 2017-10-13

**Authors:** Douglas H. Bartlett

**Affiliations:** ^1^ Center for Marine Biotechnology and Biomedicine Marine Biology Research Division Scripps Institution of Oceanography University of California San Diego La Jolla CA 92093‐0202 USA

Antibiotic resistance is generally considered to be the dark flip side to the availability of life‐saving antibacterial agents. According to the CDC, each year at least 2 million people become infected with antibiotic‐resistant bacteria, and of these, at least 23 000 perish (https://www.cdc.gov/drugresistance/index.html). However, the genes for these resistance factors also serve scientific needs. They have been used as selectable markers in recombinant DNA techniques as their inception (Cohen *et al*., 1973). It is also interesting to consider the diverse roles these genes perform outside of environments impacted by man‐made antibiotic application, where they have carried out biocontrol functions for hundreds of millions of year (Allen *et al*., 2010). Just as CRISPR/Cas systems provide clues to the evolution of phage‐prokaryote battles (Horvath and Barrangou, 2010) much could be learned of microbe–microbe communication and control if only the targets and timing of the production of their antibiotics and antibiotic resistance mechanisms were more fully understood.

Elbehery *et al*. (2016) have taken the environmental study of antibiotic resistance genes to new heights, or rather depths (Fig. 1). As reported in *Microb Biotechnol* **10** 189–202, they searched for these elements in one of the most remote and inhospitable locations on earth, a geothermally heated brine pool within the Red Sea. It is deep (2000 m), hot (68°C), salty (26%), acidic (5.3), dark, anoxic and as if that is not harsh enough, it also contains concentrations of some heavy metals about 1000× that of typical seawater. Members of the Gammaproteobacteria and Euryarchaeota dominate within this environment (Bougouffa *et al*., 2013), and viruses and mobile genetic elements have been detected in the underlying sediments (Adel *et al*., 2016).

**Figure 1:**
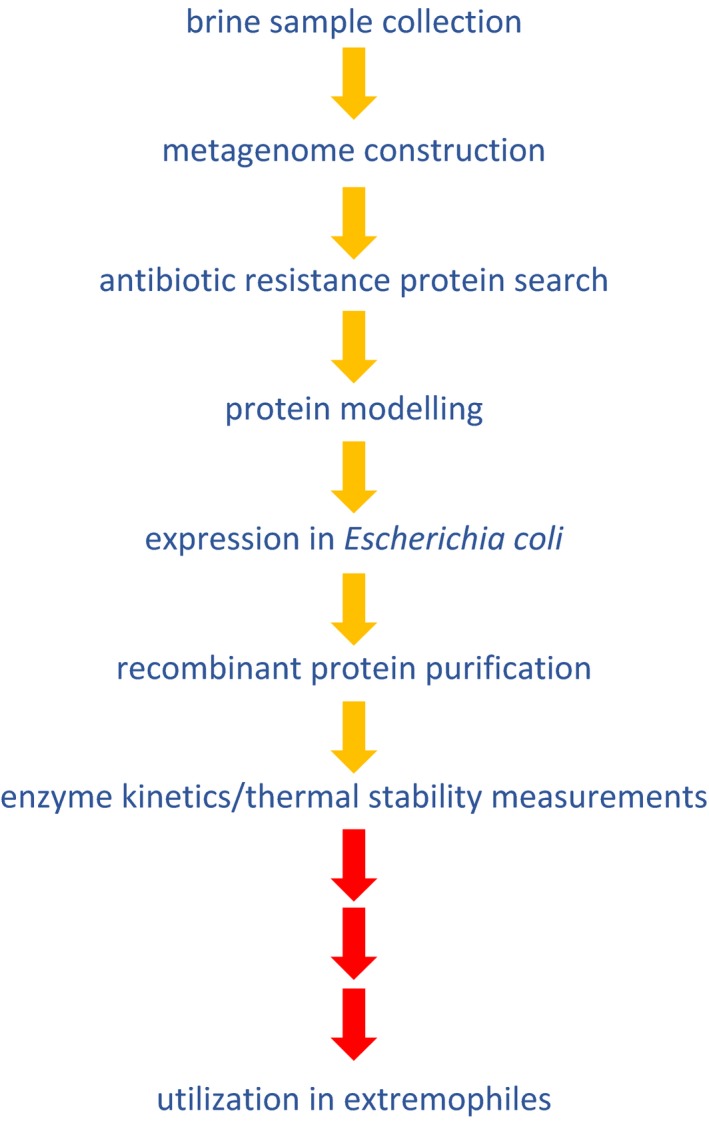
Process for novel antibiotic resistance protein discovery and characterization undertaken by Elbehery, Leak and Siam (gold arrows) and possible next steps (red arrows).

In this setting, the authors searched for antibiotic resistance genes. To do so, they aligned polypeptides translated from a Red Sea brine pool metagenome to a database of antibiotic resistance proteins. Hundreds of matches were uncovered, mostly associated with multidrug or macrolide resistance, but including many other resistance factors. A Red Sea brine beta‐lactamase‐related protein (RSB BL) and a Red Sea brine aminoglycoside kinase‐related protein (RSB AK) were selected for detailed investigation. Active sites for predicted enzyme activities were found to be conserved, and 3D modelling reinforced the predicted enzyme classifications and identified modifications possibly associated with enhanced thermal stability, such as increased numbers of salt bridges. The authors then went on to work with purified proteins produced following overexpression of the cloned genes. Enzyme kinetic and thermal stability measurements revealed exceptional characteristics in particular for the RSB AK. This included micromolar *K*_m_ values and high overall catalytic efficiencies for neomycin and kanamycin, and considerable thermal stability even at 65°C. The gene encoding RSB AK also conferred resistance to these antibiotics when expressed in *Escherichia coli*. It is the first naturally occurring thermostable member of its class. And while the deep sea contains a variety of habitats of growing interest in the search for bioactive secondary metabolites, including aminoglycosides (Tamegai *et al*., 2010; Kamjam *et al*., 2017), the examination of antibiotic resistance among microbes present at depth had not been previously pursued.

The value of this work is twofold. It presents a strategy for obtaining antibiotic resistance genes that could be used as selectable markers in thermophiles and other extremophiles. That this environment is a good place to hunt for resistance factors was previously demonstrated by the discovery of a thermotolerant and halotolerant mercury reductase derived from the same metagenome (Sayed *et al*., 2014). But, given the metal content of the brines, finding a mercury‐detoxifying enzyme, while it is interesting and obviously critical to adaptation, is perhaps not as surprising as that of the genes uncovered in the *Microbial Biotechnology* article. More importantly, this work provides a dramatic example of the evolution of antibiotic resistance far removed from the human application of these chemicals. Geothermal cooking of organics at this location does result in the abiotic synthesis of aromatic compounds, but surely these chemical alterations do not lead to the presence of complex organic structures with the finely tuned activities of microbially synthesized antibiotics (Wang *et al*., 2011). The conclusion is therefore that even in this harsh deep‐sea location, where cell numbers are reduced to 1% of those at the sea surface, microbes appear to carry out offence and defence, thrust and parry, domination and resistance. But who are the partners (Fig. 2)? What microbes produce the antibacterials in this setting and what microbes have acquired the corresponding resistance factors? And, how do these cells interact and co‐evolve with one another in a place like a Red Sea brine? Sublethal concentrations of aminoglycosides could alter quorum sensing and induce biofilm formation (Romero *et al*., 2011). So perhaps what is at play in the brines has more to do with controlling microbial community physical structure than killing microbes to remove competition or to acquire nutrients. These are all valuable questions and hypotheses that could be addressed in the future. One thing is clear. Antibiotic resistance is not just a problem, it is an opportunity to explore microbial relationships in intimate detail, even at the limits of life.

**Figure 2:**
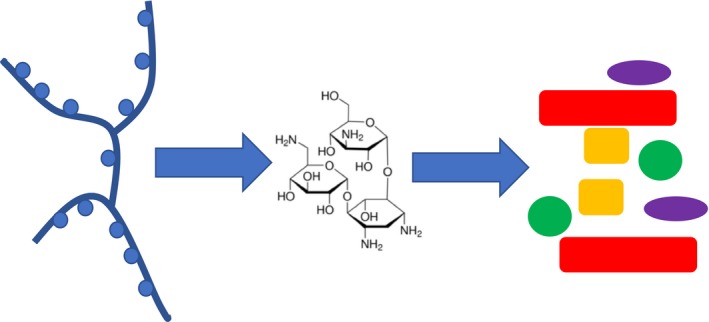
Who is talking to who? The identity and interplay of the antibiotic producers and resisters in the poly‐extreme environment of the Red Sea brine await characterization.

## Conflict of interest

None declared.
